# Dynamic Changes in Fourth Heart Sound in Type 2 Diabetes: Insights From Visualized Phonocardiography and SGLT2 Inhibitor Adjustment

**DOI:** 10.1155/carm/2871380

**Published:** 2025-08-11

**Authors:** Kunimasa Yagi, Daisuke Chujo, Shimpei Ogawa, Shumpei Saito, Makoto Iwazawa, Taketsugu Tsuchiya, Shuichi Mizuta, Naohito Yamasaki, Takashi Muro

**Affiliations:** ^1^Department of Internal Medicine, Kanazawa Medical University Hospital, Kanazawa, Ishikawa 920-0293, Japan; ^2^Center for Clinical and Translational Research, Toyama University Hospital, Toyama 930-0152, Japan; ^3^AMI Inc., 2-13 302 Higashisengokucho, Kagoshima 892-0842, Japan; ^4^Department of Trans-Catheter Cardiovascular Therapeutics, Kanazawa Medical University Hospital, Kanazawa, Ishikawa 920-0293, Japan; ^5^Department of Cardiology and Geriatrics, Kochi Medical School, Nankoku, Kochi 783-8505, Japan; ^6^Heart Valve Center, Midori Hospital, 1-16 Sumiyoshi, Nishi-ku, Kobe, Hyogo 651-2133, Japan

**Keywords:** cardiac fourth sound, sodium–glucose co-transporter-2 inhibitors, Type 2 diabetes mellitus, visualized phonocardiography

## Abstract

The fourth heart sound (S4) is an auscultatory marker of left ventricular diastolic dysfunction (LVDD). Additionally, S4 correlates with atrial function, which is typically impaired in patients with Type 2 diabetes (T2D) but can improve with sodium–glucose co-transporter-2 inhibitor (SGLT2i) therapy. This case report highlights the dynamic changes in S4 associated with modification of SGLT2i therapy. An 83-year-old male with T2D and LVDD, confirmed via echocardiography, was treated with SGLT2i therapy for 4 years for glycemic control. The therapy was discontinued in December 2023 because of increased nocturnal urination. Two months after discontinuation, the patient developed pronounced S4, accompanied by mild chest discomfort and worsening of evening leg edema. Resumption of SGLT2i therapy led to a marked reduction in S4 along with a remarkable improvement in chest discomfort and edema within 1 month. These findings were confirmed by visual phonocardiography. This case underscores the potential utility of dynamic S4 changes as a noninvasive indicator of SGLT2i therapy adjustment. These findings highlight the novel clinical application of S4 monitoring in mitigating heart failure progression in patients with T2D.

## 1. Introduction

Asymptomatic left ventricular diastolic dysfunction (LVDD), a common precursor to heart failure with preserved ejection fraction (HFpEF) [1], is highly prevalent [2] and increasingly observed in patients with Type 2 diabetes (T2D) [3]. Sodium–glucose co-transporter-2 inhibitors (SGLT2i) are well-established treatments for managing T2D and HFpEF [4].

The fourth heart sound (S4), an atrial sound that occurs just before S1, is dull, low-pitched, and critically associated with LVDD [5]. S4 indicates increased resistance during left ventricular filling following left atrial contraction [6]. Its amplitude increases with myocardial stiffness and represents the atrial kick [7]. Therefore, S4 serves as a valuable marker for assessing LVDD and atrial function, both of which are frequently impaired in patients with T2D [8, 9].

Herein, we present the case of a patient with T2D and elevated septal E/E′ who was treated with SGLT2i for more than 4 years. This case highlights the dynamic changes in S4 associated with SGLT2i adjustment, underscoring the potential utility of S4 assessment in managing patients with T2D.

## 2. Case Report

An 83-year-old male with a 15-year history of T2D, hypertension, and dyslipidemia was treated at an outpatient clinic. The clinical characteristics of the patients are presented in Table 1 blood pressure 125/68 mmHg and HbA1c 6.5% in December 2023. The patient had no diabetic complications, except stage 2 diabetic nephropathy (G3aA2). Coronary artery disease was ruled out using cardiac computed tomography in 2012, double-master electrocardiography in 2021, and the absence of regional hypokinesis on transthoracic echocardiography (TTE) in May 2019. TTE findings, including a left ventricular ejection fraction (LVEF) of 59%, E/A ratio of 0.70, E velocity of 67 m/s, septal E/E′ of 16, and septal E′ of 4.13, were compatible with LVDD [10].

The patient was treated with 10 mg empagliflozin for glycemic management and had an HbA1c level of 7.0% for over 4 years until the last week of December 2023. The patient requested discontinuation of SGLT2i therapy because of frequent nocturnal urination, possibly related to subclinical prostatitis. As the patient's HbA1c level was maintained below 7.0% with the combination of SGLT2i and repaglinide, discontinuation of SGLT2i therapy was agreed upon.

**Figure 1:**
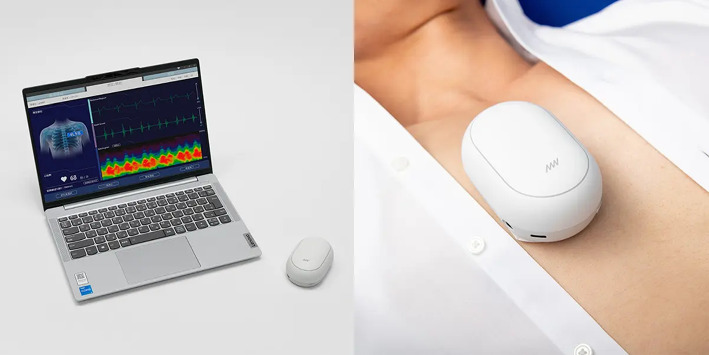
Visualized phonocardiography, the AMI-SSS01 series. The AMI-SSS01 series (AMI Inc., Kagoshima, Japan), which is approximately the size of a computer mouse, was designed for patients in the supine position. The bipolar-lead ECG signals were collected through electrodes located on its bottom surface, ensuring direct contact with the skin. The heart sounds were simultaneously captured using a microphone embedded within the device. The collected heart-sound signals were amplified using an operational amplifier and audio preamplifier. The signals were digitized using an analog-to-digital converter at a sampling rate of 8 kHz. The digitized signals were processed every 2 ms into packets containing 16 ECG samples and one phonocardiogram sample. Data packets were transmitted to a PC monitor for analysis and visualization. The AMI-SSS01 series have undergone rigorous evaluation and received clearance from the Pharmaceuticals and Medical Devices Agency (PMDA), Japan's regulatory institution. This approval followed stringent official testing procedures, ensuring the reliability of the device and compliance with the safety standards. ECG, electrocardiogram.

**Figure 2:**
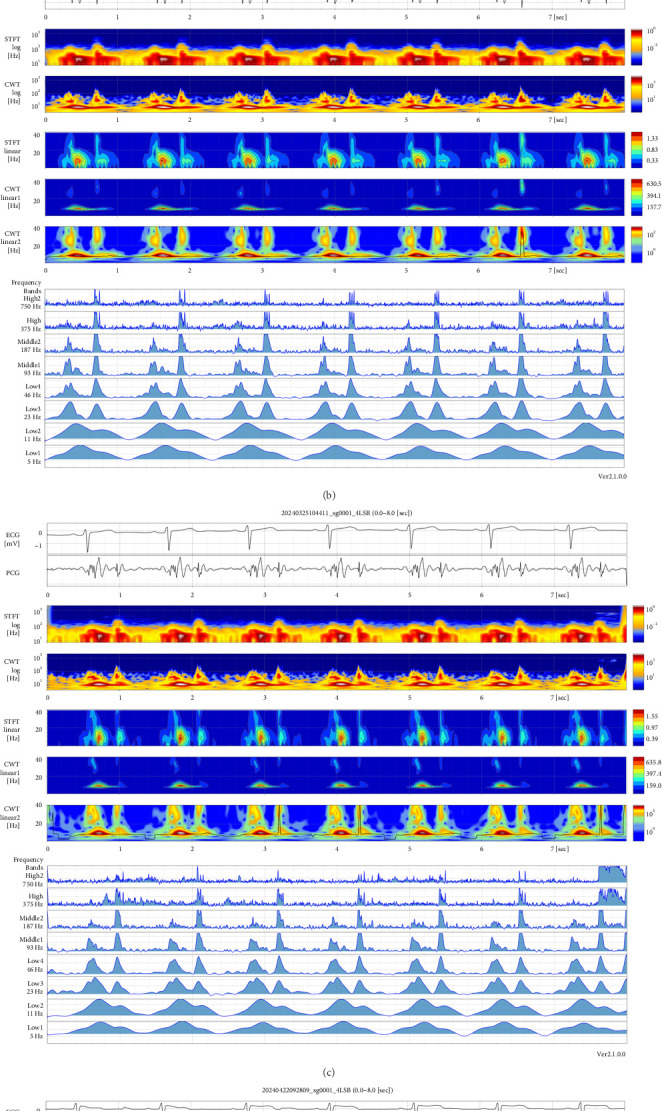
Phonocardiograms recorded at 4LSB were visualized using two frequency analysis methods—STFT and CWT—in the final week of (a) December 2023, (b) January 2024, (c) March 2024, and (d) April 2024. The filter band CWT indicates the frequency band. 4LSB, fourth left sternal border; CWT, continuous wavelet transformation; ECG, electrocardiogram; Hz, hertz; PCG, phonocardiogram; STFT, short-time Fourier transformation; sysM, systolic murmur.

Cardiac auscultation findings were evaluated using a traditional stethoscope and phonocardiography with a PMDA-approved AMI-SSS01 series (AMI Inc., Kagoshima, Japan) (Figure 1). Heart sounds recorded at the fourth left sternal border (4LSB) are depicted in Figures 2(a), 2(b), 2(c), 2(d), and those at the fifth left midclavicular line (5LMCL) are presented in Supporting Figure 1a–1d. Routine consultations in late January (Figure 2(b)) and March 2024 (Figure 2(c)) revealed the presence of S4, systolic murmur, and accentuated second heart sound (S2). The presence of S4 was confirmed by its characteristic appearance as a wave separated from S1, occurring before S1 in short-time Fourier transformations (STFT) and continuous wavelet transformations (CWT). Alternatively, it was visualized as a notch on the ascending limb of the S1 peak within low-frequency bands.

The patient also experienced mild chest discomfort and lower leg edema in the evening. Two months after discontinuing SGLT2i therapy, the patient consented to resume the treatment. This led to a marked improvement in symptoms, including resolution of chest discomfort and reduction in edema. A follow-up TTE in October 2024 showed findings comparable to those prior to discontinuation (LVEF, 55%; E/A ratio, 0.56; septal E/E′, 19; and septal E′, 2.5), indicating no significant change in diastolic function based on conventional indices.

In contrast, visualized phonocardiography demonstrated the disappearance of S4 (Figure 2(d)). This finding, together with symptomatic improvement, suggests that S4 may serve as a sensitive and noninvasive marker of subtle hemodynamic changes that may not be reflected in standard echocardiographic parameters. These observations support the potential clinical utility of SGLT2i therapy not only in glycemic control but also in preserving diastolic function in patients with T2D.

## 3. Discussion

### 3.1. Interpretation of Findings

In this case, we observed an enhancement of S4 following the discontinuation of SGLT2i therapy, with a subsequent reduction upon its re-administration. These observed dynamic changes in S4 were objectively confirmed using visual phonocardiography, highlighting their potential as real-time markers of LVDD and atrial function, offering a new perspective for early assessment of diastolic impairment and treatment response.

S4, an auscultatory indicator of LVDD, is generated by compensatory left atrial contraction during late diastole, corresponding to the atrial kick and Doppler echocardiographic A-wave [6]. The amplification or attenuation of S4 reflects shifts in the hemodynamic status, offering insights into myocardial and atrial performance. By alleviating volume overload, SGLT2i therapy may reduce S4 occurrence, even under physical stress. Additionally, left atrial dysfunction, frequently observed in patients with T2D [8, 9], improves with prolonged SGLT2i therapy [11, 12].

The cardioprotective effects of SGLT2i are multifaceted, including a reduction in cardiac workload, improving hemodynamics through diuresis-driven volume reduction, enhancing myocardial metabolism via increased ketone body production, and improving oxygen delivery through elevated hematocrit levels [13]. Collectively, these mechanisms mitigate the risk of heart failure onset and progression.

In this patient, the abrupt discontinuation of a 4-year SGLT2i regimen likely disrupted the left atrial function, resulting in pronounced atrial contraction and a prominent S4. These findings underscore the critical role of SGLT2i therapy in maintaining hemodynamic stability and optimizing atrial performance in patients with T2D.

### 3.2. Clinical Implications

The clinical focus on S4 arises from the lack of reliable and objective markers for detecting early-stage LVDD in patients with T2D. Early identification of LVDD is clinically meaningful, as it informs therapeutic decisions, including the selection of antidiabetic agents with proven efficacy in preventing heart failure progression [14]. Although BNP has been associated with long-term LVDD progression in patients with T2D [15], and BNP is recognized as a gold-standard biomarker for cardiac function and is recommended in current heart failure guidelines [16], it has several well-documented limitations. Its levels can be influenced by extracardiac factors such as high body mass index and insulin resistance, both of which are common in T2D and may lead to underestimation of disease severity [17].

In the present case, BNP levels remained largely unchanged despite clinically apparent fluctuations in symptoms following the withdrawal and re-initiation of SGLT2i therapy. In contrast, S4 exhibited clear dynamic changes that paralleled symptomatology, suggesting its potential utility as a sensitive and responsive marker for detecting subclinical hemodynamic shifts.

Unlike BNP, S4 can be assessed noninvasively and repeatedly using only a stethoscope, without the need for blood sampling. While conventional auscultation has limitations due to subjectivity and dependence on clinician experience, these concerns can be addressed through visualized phonocardiography. This approach enhances objectivity, allows for consistent detection of subtle acoustic changes, and reduces interoperator variability [18]. The visualized phonocardiography system used in this study (AMI-SSS01series) captures heart sounds via a microphone and produces wavelet-based visual representations using STFT and CWT. This technology enables reproducible, quantitative, and temporal comparisons of heart sounds, thus broadening the clinical applicability of S4 assessment.

The complementary nature of S4 and BNP assessment may be particularly beneficial when BNP results are incongruent with clinical findings. In such cases, S4 evaluation may provide an additional physiological perspective, supporting more nuanced clinical judgment. This case underscores the potential value of incorporating S4 monitoring, particularly with visualized phonocardiography, into the routine assessment of cardiac function in patients with T2D. Despite its noninvasive and repeatable nature, the role of S4 in diabetes care remains underutilized. We believe this report offers an important step toward validating S4 as a complementary diagnostic tool alongside BNP, and it may serve as a foundation for future investigations in this field.

### 3.3. Limitations

This case report has several limitations. First, the observation period was short, and long-term follow-up data were not available. A longer observation period would be essential to fully elucidate the relationship between changes in S4 and cardiac function following SGLT2i therapy. To our knowledge, no prior studies have investigated heart sounds—including S4—in patients with diabetes or those treated with SGLT2i. Nevertheless, this case demonstrates that marked changes in S4 can occur even in elderly individuals whose lifestyle is unlikely to have changed significantly over a short period. These findings suggest the feasibility and potential clinical relevance of S4 assessment in patients undergoing SGLT2i therapy.

Second, the use of TTE was limited. In regional core hospitals, where demand for TTE is high and human resources are limited, priority is often given to more severe cases. As a result, repeated or urgent echocardiographic evaluations may not be feasible. Furthermore, strain imaging, which could have provided deeper insight into myocardial and atrial function, was not performed. This reflects a common issue in many clinical settings, where facilities lack both the equipment and specialized personnel needed for detailed atrial function assessment.

Finally, visualized phonocardiography, although promising, is not yet widely recognized or adopted in global clinical practice. However, its low cost, ease of use, and noninvasive nature make it an attractive option, particularly in resource-constrained settings. This case underscores the potential of visualized phonocardiography as a practical alternative for monitoring diastolic function in real-world clinical environments.

## 4. Conclusion

This case highlights the dynamic changes in S4 associated with the discontinuation and re-administration of SGLT2i, demonstrating its potential as a real-time marker of LVDD and atrial function in T2D. Unlike conventional markers, such as BNP, S4 offers immediate insights into hemodynamic shifts, underscoring the utility of visualized phonocardiography in diabetes management. Given its accessibility and cost-effectiveness, this technology may be pivotal in optimizing therapeutic strategies and evaluating outcomes, particularly in resource-limited settings.

## Figures and Tables

**Table 1:** Course of heart sounds and other clinical parameters.

Events	December 2023 last week	January 2024 last week	March 2024 last week	April 2024 last week
	SGLT2i discontinuation		SGLT2i reintroduction	
BW (kg)	61.4	62.3	61.7	59.7

BMI (kg/m^2^)	24.0	24.3	24.1	23.3

sBP (mmHg)	125	147	148	140

dBP (mmHg)	68	73	83	80

HbA1c (%)	6.5	6.8	6.4	6.3

GA (%)	15.9	17.2	15.2	14.3

PG (mg/dL)	131	125	138	101

BNP (pg/mL)	69.7	67.3	57.4	57.4

Chest discomfort in the evening	(−)	(+)	(+)	(−)

Edema	(±)	(++)	(++)	(+)

S4 in 4LSB	(−)	(+)	(+)	(−)

S2 in 4LSB	(+)	(++)	(++)	(++)

sysM in the 4LSB	(−)	(++)	(++)	(+)

S4 in 5LMCL	(−)	(++)	(+)	(−)

S2 in 5LMCL	(+)	(++)	(++)	(+)

sysM in 5LMCL	(−)	(+)	(++)	(+)

Other medications	Pitavastatin 1 mg/d	Pitavastatin 1 mg/d	Pitavastatin 1 mg/d	Pitavastatin 1 mg/d
	Repaglinide 0.75 mg/d	Repaglinide 0.75 mg/d	Repaglinide 0.75 mg/d	Repaglinide 0.75 mg/d

*Note:* GA, glycoalbumin; S2, second heart sound; S4, fourth heart sound; SGLT2i, sodium–glucose co-transporter-2 inhibitors; sysM, systolic murmur.

Abbreviations: 4LSB, fourth left sternal border; 5LMCL, fifth left midclavicular line; BMI, body mass index; BNP, brain natriuretic peptide; BW, body weight; dBP, diastolic blood pressure; PG, plasma glucose; sBP, systolic blood pressure.

## Data Availability

The data that support the findings of this study are available from the corresponding author upon reasonable request.

## Nomenclature

S4: The cardiac fourth heart sound
S2: The cardiac second heart sound
SGLT2i: Sodium–glucose co-transporter-2 inhibitor
HFpEF: Heart failure with preserved ejection fraction
LVDD: Left ventricular diastolic dysfunction
TTE: Transthoracic echocardiography
BNP: B-type natriuretic peptide
NP: Natriuretic peptide
STFT: Short-time Fourier transformations
CWT: Continuous wavelet transformations

## Supporting Information

Supporting Figure 1 shows the record of heart sounds at 5LMCL.
