# Supplementary material for: Dynamic Changes in Fourth Heart Sound in Type 2 Diabetes: Insights From Visualized Phonocardiography and SGLT2 Inhibitor Adjustment
Source: Case Rep Med. 2025 Aug 11;2025:2871380. doi: 10.1155/carm/2871380 (PMC12360888; doi:10.1155/carm/2871380)
Supplement: Supporting Information — Additional supporting information can be found online in the Supporting Information section. [file 2871380.f1.docx]

**Supplementary Figure 1. Record of heart sounds at 5LMCL**

S1a) 5LMCL: December, 2023


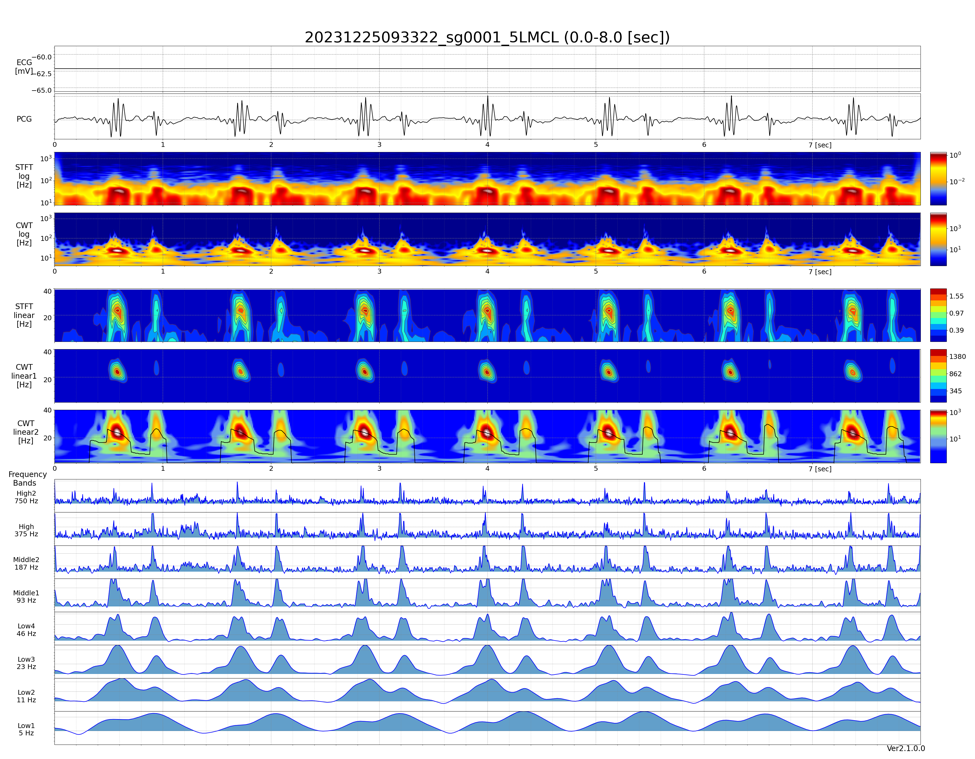


S1b) 5LMCL: January, 2024


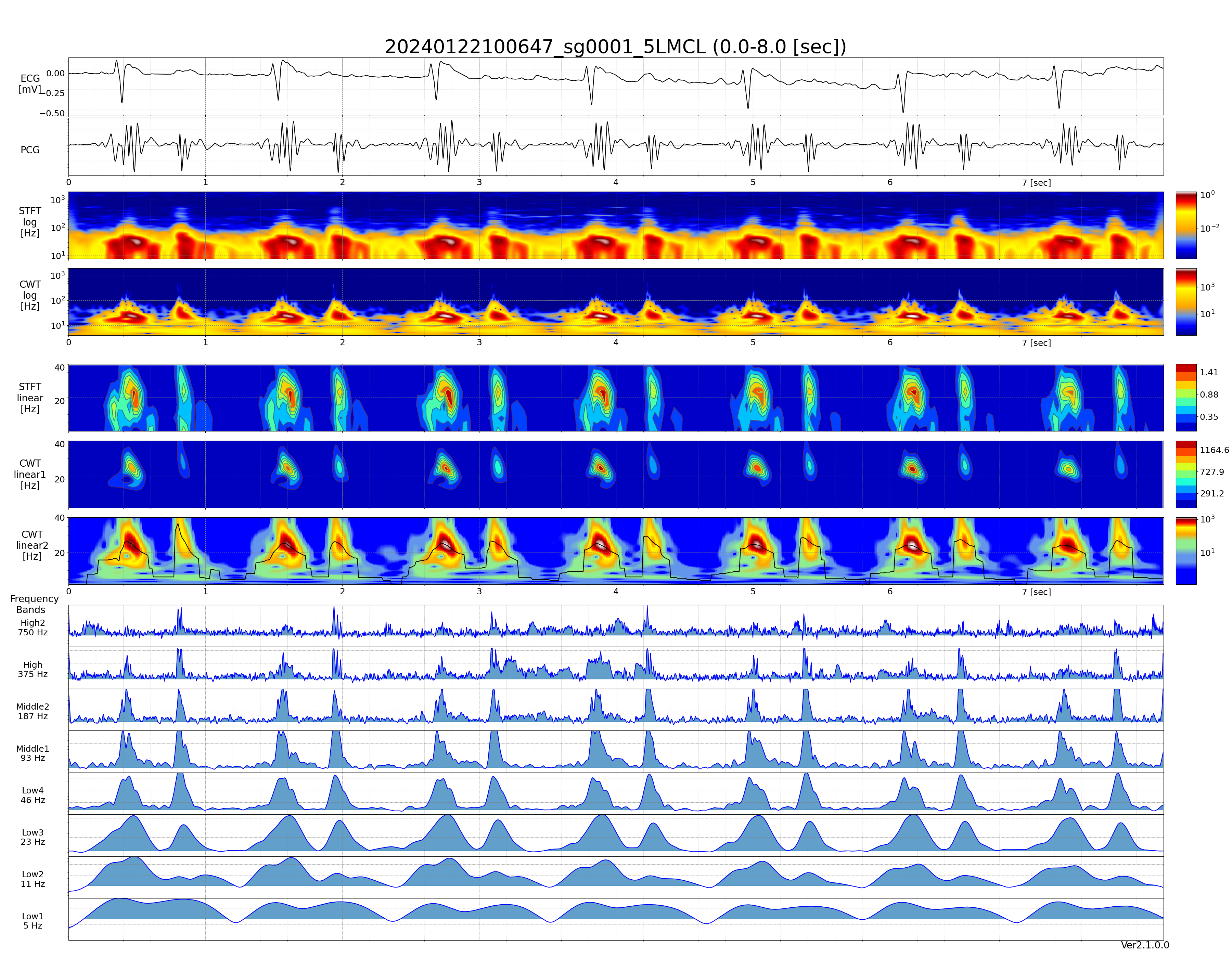


S1c) 5LMCL: March, 2024


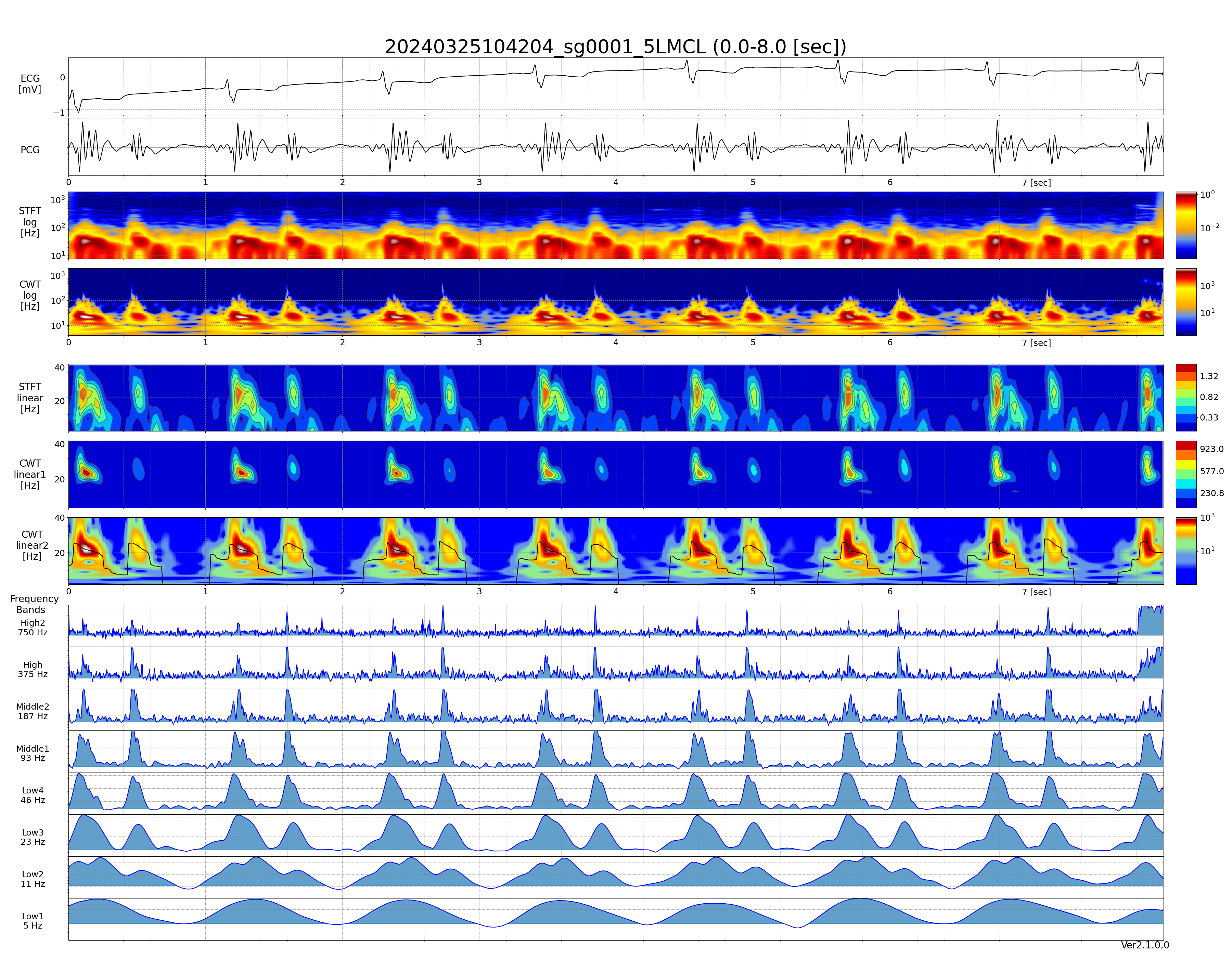


S1d) 5LMCL: April, 2024


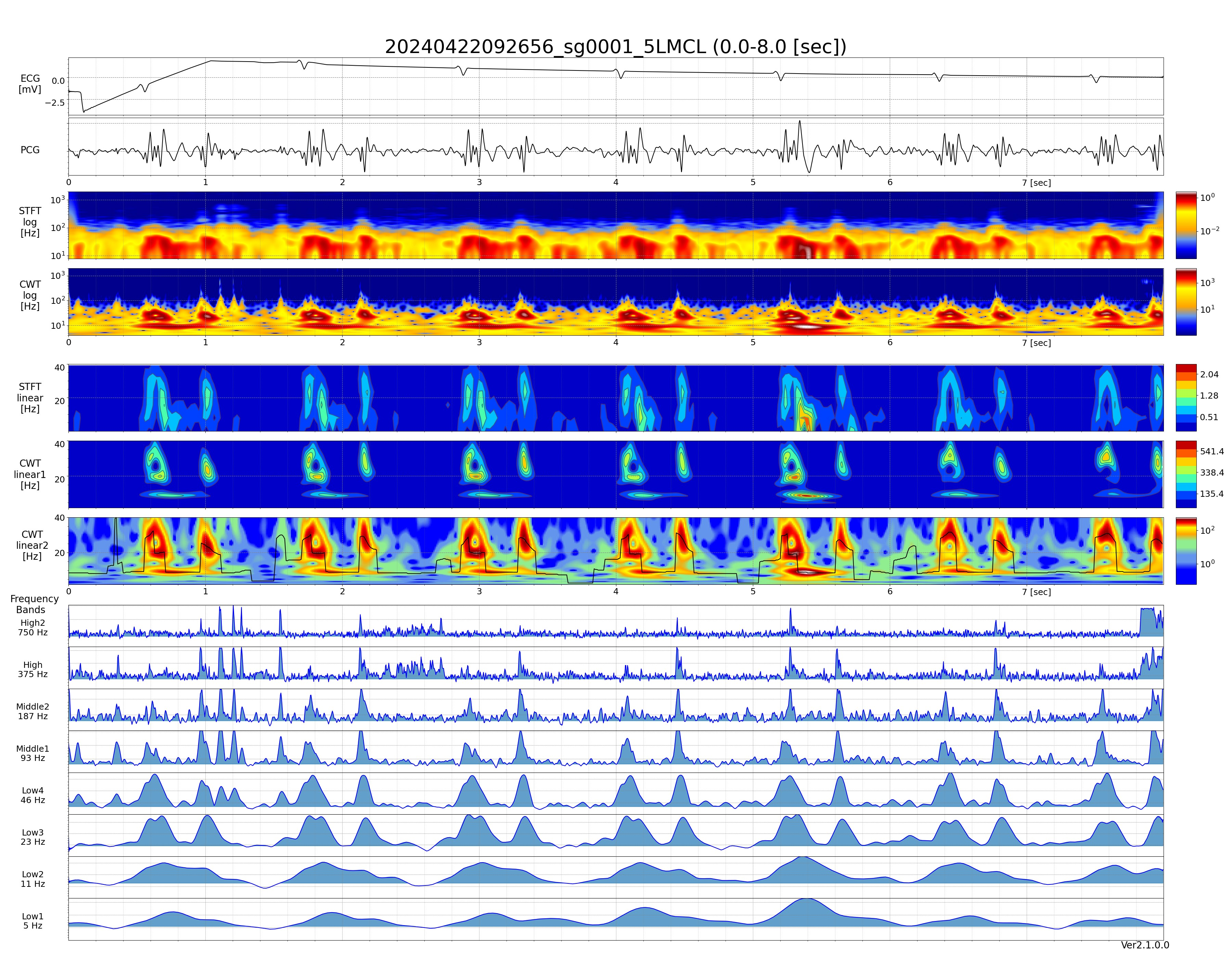


Visualized phonocardiograms were recorded in the last weeks of (a) December 2023, (b) January 2024, (c) March 2024, and (d) April 2024 using two methods of frequency analysis of heart sounds (STFT and CWT). The frequency bands are indicated by the CWT filter bank.

5LMCL, fifth left midclavicular line; CWT, continuous wavelet transformation; ECG, electrocardiogram; Hz, Hz; PCG, phonocardiogram; S1, first heart sound; S2, second heart sound; S4, fourth heart sound; STFT, short-term Fourier transformation.
